# Discrete papular lichen myxedematosus successfully treated with isotretinoin

**DOI:** 10.1016/j.jdcr.2025.10.028

**Published:** 2025-10-27

**Authors:** Alexa Figueroa Baiges, Nina Punyamurthy, Alexander M. Maley

**Affiliations:** a University of Wisconsin School of Medicine and Public Health, Madison, Wisconsin; b Department of Dermatology, Medical College of Wisconsin, Milwaukee, Wisconsin; c Department of Dermatology, Aurora St. Luke’s Medical Center, Milwaukee, Wisconsin

**Keywords:** discrete papular lichen myxedematosus, isotretinoin, mucinoses, scleromyxedema

## Introduction

Discrete papular lichen myxedematosus (DPLM) is a rare subtype of cutaneous mucinosis characterized by asymptomatic 2-5 mm, firm, waxy, flesh-colored papules that are primarily distributed on the extremities and trunk without skin induration.^1^ There are few published reports of successful treatment of this condition. Previous publications describe variable responses to topical corticosteroids, intralesional steroids, tacrolimus, and electrocauterization.^2^ We present a case of DPLM in a 48-year-old man successfully treated with low-dose oral isotretinoin.

## Case report

**Fig 1:**
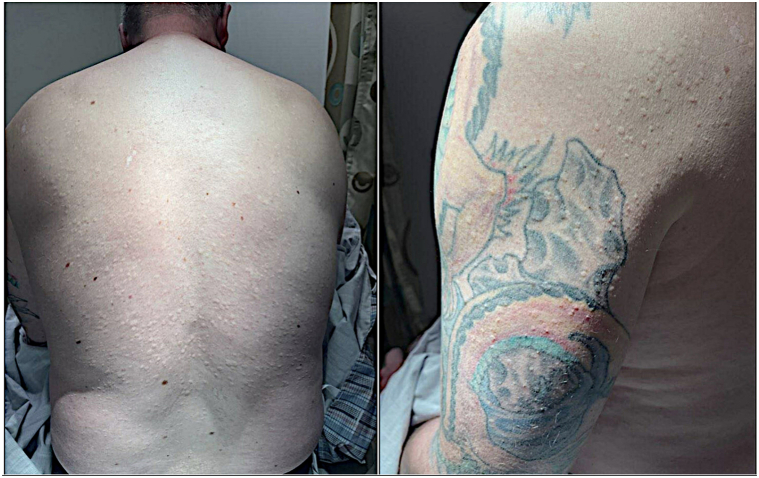
Hypopigmented to skin-colored papules on the back and left shoulder at presentation.

A 48-year-old man presented with asymptomatic lesions on his back that had been progressively appearing since his 30s. Physical examination revealed numerous oval, flesh-colored papules scattered across the upper and mid-back, posterior shoulders, and lateral arms (Fig 1).

**Fig 2:**
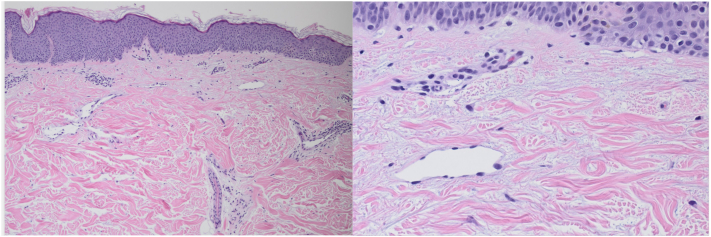
Histopathology of a punch biopsy demonstrated increased mucin deposition in the upper dermis between collagen bundles.

This had been previously diagnosed as tinea versicolor by his primary care physician and treated with ketoconazole shampoo and nystatin cream without improvement. Histological examination of a punch biopsy from the left shoulder demonstrated increased mucin deposition in the upper dermis and between deep dermal collagen bundles (Fig 2). An elastin stain was performed to rule out pseudoxanthoma elasticum, and a Giemsa stain was done to rule out urticaria pigmentosum.

Laboratory evaluation revealed mildly elevated liver enzymes (AST 41, ALT 75); complete blood count, renal function, thyroid function, ANA, and serum protein electrophoresis were within normal limits. The patient had no risk factors for hepatitis or HIV infection.

**Fig 3:**
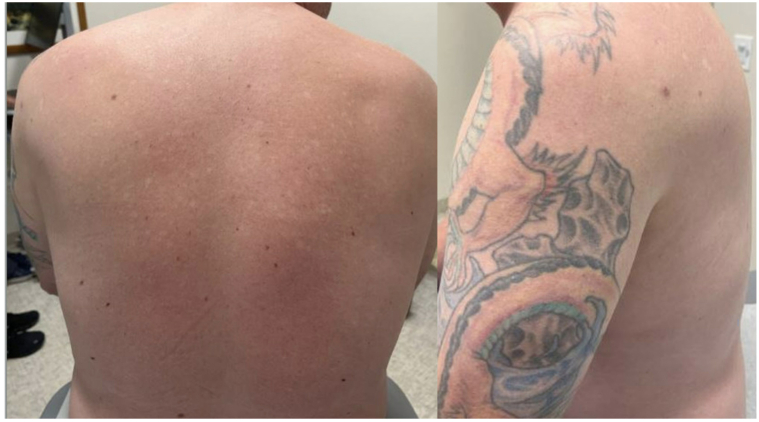
Back and left shoulder after 4 months of isotretinoin.

A diagnosis of DPLM was made based on clinical, histological, and laboratory findings. He was started on isotretinoin 40 mg daily (0.4 mg/kg). At his 1-month follow-up visit, he demonstrated marked improvement with a significant reduction in papules. By 2 months, the majority of the papules on the shoulders, upper back, and arms had resolved, with only a few remaining lesions confined to the mid-back. At 4 months, only scattered hypopigmented macules remained on the back (Fig 3), and the patient expressed high satisfaction with the results.

His liver enzymes normalized when rechecked after 2 months of therapy. He completed a 5-month course of isotretinoin (total cumulative dose 61 mg/kg). Ten months after completing isotretinoin, he has had no recurrence.

## Discussion

Lichen myxedematosus (LM) encompasses a spectrum of primary cutaneous mucinoses characterized by dermal mucin deposition and fibroblast proliferation. This condition is classified into 3 broad clinical subtypes: scleromyxedema (generalized LM), localized LM, and atypical variants. Scleromyxedema is a disorder characterized by a widespread eruption of firm, waxy papules and areas of induration due to dermal mucin deposition. It is an uncommon disease typically associated with monoclonal gammopathy and systemic manifestations. Diagnostic criteria include (1) a generalized papular and sclerodermoid eruption, (2) histopathologic findings of mucin deposition, fibroblast proliferation, and fibrosis (microscopic triad), or an interstitial granulomatous pattern, (3) the presence of monoclonal gammopathy, and (4) the absence of thyroid dysfunction.^1^ Systemic involvement is common and may affect the neurologic, gastrointestinal, pulmonary, and cardiovascular systems.^3^ Mortality in scleromyxedema is primarily related to the progression of the underlying monoclonal gammopathy to multiple myeloma and complications from internal organ involvement. Among the most severe complications is scleromyxedema-associated encephalopathy, a rare but potentially fatal condition characterized by fever, confusion, seizures, and coma. Other life-threatening sequelae include restrictive cardiomyopathy, interstitial lung disease, and dysmotility of the gastrointestinal tract.^3^ Early recognition and systemic treatment are therefore critical to prevent irreversible end-organ damage.

In contrast, localized variants of LM are limited to the skin and lack systemic findings. There are 5 subtypes of localized LM: DPLM, acral persistent papular mucinosis, cutaneous mucinosis of infancy, self-healing papular mucinosis, and nodular LM.^3^^,^^4^ These lesions are typically confined to a specific region of the body and do not exhibit sclerodermoid changes. Distinguishing scleromyxedema from its localized counterparts is essential, as the former carries a significantly higher risk of systemic morbidity and mortality, necessitating a more aggressive diagnostic and therapeutic approach.

DPLM, as in our case, presents with discrete, waxy papules, primarily involving the trunk and extremities. It is characterized by excessive dermal mucin deposition and fibroblast proliferation. It is an exceedingly rare diagnosis, with only generating 14 non-HIV DPLM cases reported and only 2 cases describing successful treatment with corticosteroids (Table I). The pathogenesis remains poorly understood, and no standardized treatment exists. There have been few reports of treatment for the diffuse papular type of LM with varying rates of success. These include topical steroids, intralesional steroids, topical tacrolimus, and electrocauterization. Reported therapies across all variants of LM include excision, dermabrasion, CO_2_ laser, and intralesional corticosteroids or hyaluronidase, with mixed success. Few reports of LM describe systemic therapy. Isotretinoin has been previously used in cases of scleromyxedema^17^^,^^18^ and follicular mucinosis,^19^ suggesting potential efficacy in mucinous dermatoses.

**Table I:** Reported cases of non-HIV-related discrete papular lichen myxedematosus

First author, year	Patient demographics	Treatment	Response
Woerdeman, 1960^5^	19-y-old man	None reported	NA
Tay, 1970^6^	41-y-old man	Oral triamcinolone, Oral thyroxine	No improvement
Enerbach, 1976^7^	46-y-old man	Topical steroids	NA
Coskey, 1977^8^	22-y-old man	None reported	NA
Abd El-Aal, 1981^9^	32-y-old woman	None reported	NA
Poswig, 2000^2^	62-y-old man	None reported	NA
Rongioletti, 2001^10^	2 males, 1 female	None reported	NA
Bragg, 2008^11^	56-y-old woman	None reported	NA
Concheiro, 2009^12^	21-y-old woman	None reported	NA
Hadj, 2014^4^	42-y-old man	Topical steroids	Good improvement
Tam, 2014^13^	23-y-old man	Fluocinonide 0.05% twice a day, intralesional triamcinolone once monthly × 3 mo	Resolution of most lesions, significant flattening of the rest
Christman, 2017^14^	62-y-old woman	Topical tacrolimus 0.1%	Not reported
Garber, 2017^15^	57-y-old woman	Topical tacrolimus 0.1%	No improvement
Saenz-Aguirre, 2020^16^	42-y-old man	Clobetasol 0.05%, tacrolimus 0.01%, electrocauterization	No improvement

*NA*, Not available.

Isotretinoin is thought to exert therapeutic effects in LM by modulating fibroblast activity and mucin production, both central to disease pathophysiology. At the molecular level, it influences gene expression related to cell cycle regulation and differentiation, leading to normalization of epidermal turnover and suppression of fibroblast proliferation. This cascade results in reduced dermal mucin deposition and clinical improvement. Additionally, isotretinoin possesses anti-inflammatory and immunomodulatory properties that may further enhance therapeutic outcomes.^20^ Our patient achieved complete clearance after 5 months of 40 mg of isotretinoin; dose escalation or extended therapy was not required due to rapid clinical improvement achieved.

This case highlights the potential role of low-dose isotretinoin as a safe and effective treatment option for DPLM, with rapid improvement and long-lasting results.

## Conflicts of interest

None disclosed.
